# Changes in nonalcoholic fatty liver disease and M2BPGi due to lifestyle intervention in primary healthcare

**DOI:** 10.1371/journal.pone.0298151

**Published:** 2024-02-29

**Authors:** Eun-Hee Nah, Yong Jun Choi, Seon Cho, Hyeran Park, Suyoung Kim, Eunjoo Kwon, Han-Ik Cho

**Affiliations:** 1 Department of Laboratory Medicine, Chonnam National University Hwasun Hospital, Hwasun, South Korea; 2 Health Promotion Research Institute, Korea Association of Health Promotion, Seoul, South Korea; 3 MEDIcheck LAB, Korea Association of Health Promotion, Seoul, South Korea; Kaohsiung Medical University Hospital, TAIWAN

## Abstract

**Background:**

A healthy lifestyle is the most important method for managing nonalcoholic fatty liver disease (NAFLD). Mac-2-binding protein glycosylated isomer (M2BPGi) has been suggested as a biomarker for NAFLD. This study aimed to determine the efficacy of personalized lifestyle interventions on NAFLD remission.

**Methods:**

This single-arm intervention study recruited participants with NAFLD who underwent health checkups at seven health-promotion centers in five South Korean cities. Fatty liver diagnosis was based on ultrasonography (US). The 109 individuals were recruited for personalized lifestyle interventions of hypocaloric diets and exercise. The participants attended the lifestyle intervention programs once per month for the first 3 months, and once every 3 months for the subsequent 6 months. In addition to sessions through center visits, phone-based intervention and self-monitoring at 4-, 5-, 7-, and 8-month were provided during the 9-month intervention period. And phone-based self-monitoring were also provided monthly during the 3-month follow-up period. The primary outcome was NAFLD remission at month 12 as measured on US and magnetic resonance elastography. The secondary outcomes were the changes in metabolic factors and M2BPGi.

**Results:**

The 108 individuals (62 males and 46 females; age 51.1±12.4 years, mean±standard deviation) were finally analyzed after the 12month intervention. Body mass index, waist circumference (WC), blood pressure, blood lipids (total cholesterol, triglycerides, and HDL-C), and fasting blood sugar levels were improved relative to baseline (all *P*<0.05). Fatty liver at or above the moderate grade according to US was decreased at month 12 relative to baseline (67.6% vs 50.9%) (*P* = 0.002). M2BPGi levels decreased during the 12-month study period (*P*<0.001). M2BPGi levels were moderately correlated with hepatic fat fraction by magnetic resonance imaging (*r* = 0.33, *P* = 0.05). WC (OR = 0.82, 95% CI = 0.67–1.00, *P* = 0.05) and HDL-C (OR = 1.17, 95% CI = 1.03–1.32, *P* = 0.014) were associated with remission of fatty liver in the multivariate analysis.

**Conclusion:**

The personalized lifestyle intervention was effective in improving fatty liver and metabolic factors, but not hepatic stiffness, in NAFLD.

**Trial registration:**

ICTRP, cris.nih.go.kr (KCT0006380).

## Introduction

The increasing prevalence of obesity and diabetes is resulting in nonalcoholic fatty liver disease (NAFLD) also becoming increasingly prevalent. Approximately 25% of the worldwide population was estimated to have NAFLD [1], affecting 60% of patients with diabetes [2] and 90% of people with obesity [3]. The spectrum of NAFLD ranges from simple steatosis to nonalcoholic steatohepatitis (NASH). Patients with NAFLD, and especially those with NASH, may eventually progress to fibrosis, leading to cirrhosis and hepatocellular carcinoma. The fibrosis stage of liver disease is known to be associated with the long-term outcomes in individuals with NAFLD [4, 5].

NAFLD development is related to lifestyle factors such as a high-calorie diet with reduced physical activity and exercise. Effectively treating NASH is required to interrupt the disease progression [6]. Lifestyle changes that focus on weight loss remain the cornerstone of NASH treatment [7]. Lifestyle interventions combined with a 10% decrease in body weight may improve the states of steatosis, inflammation, and even fibrosis [8]. Recent studies were conducted at a tertiary medical center and expert centers [8, 9]. Some studies used interventions that involved tightly controlled diets over short periods ranging several weeks to months [10–12]. However, it is unlikely that participants can adhere to such diets for a long time to achieve NAFLD remission, and hence a community-based lifestyle modification program that could be applied in primary healthcare centers is needed.

Imaging technologies such as ultrasonography (US), magnetic resonance imaging (MRI), and transient elastography have also been widely used for the assessment of liver steatosis and stiffness. Magnetic resonance elastography (MRE) has been demonstrated to show high diagnostic accuracy for liver fibrosis [13, 14]. An MRI-based technique of measuring the proton-density fat fraction (PDFF) was developed to measure the hepatic fat level. This technique can accurately measure the fat levels of all hepatic areas [15, 16]. However, considering its cost and need for MRI equipment, it is inappropriate to apply these techniques to periodic follow-ups of the degree of hepatic fat and fibrosis in individuals with NAFLD in primary healthcare. Therefore, simple, reliable, and noninvasive biomarkers also need to be identified for assessing the change in hepatic steatosis and fibrosis in NAFLD. This study aimed to determine the efficacy of personalized lifestyle interventions on NAFLD remission, and on the improvement of metabolic factors and Mac-2-binding protein glycosylated isomer (M2BPGi) in primary healthcare.

## Materials and methods

### Study design and participants

This single-arm intervention study had a prospective multicenter design. Participants were recruited from among health examinees who underwent health checkups at seven health-promotion centers in five South Korean cities. The recruitment began approximately one month before the planned start of the intervention from June 3, 2021 to June 28, 2021. The inclusion criteria were as follows: aged 20–80 years, body mass index (BMI) of ≥25 kg/m^2^, fatty liver of at least mild severity as measured by abdominal US, and no history of viral hepatitis or hepatocellular malignancy, secondary causes of fatty liver, or high alcohol consumption (>210 g for males and > 140 g for females weekly).

All subjects provided written informed consent. The authors confirm that all related trials for this intervention are registered. This study protocol was reviewed and approved by the Institutional Review Board of the Korea Association of Health Promotion in April 15, 2021 (approval no. 130750-202104-HR-004), and the study was prospectively registered with the Korean Clinical Trials Registry at ICTRP, cris.nih.go.kr (KCT0006380).

### Intervention & follow-up

These lifestyle interventions were implemented between July 2, 2021 and July 30, 2022. Dietitians at seven health-promotion centers were instructed about the operating protocol and provided with a manual to ensure that the trial was standardized. Participants received six sessions (at baseline, and after 1, 2, 3, 6, and 9 months) at visits to the health promotion centers over 9 months. In addition to sessions through center visits, phone-based intervention and self-monitoring at 4-, 5-, 7-, and 8-month were provided during the 9-month intervention period. And phone-based self-monitoring were also provided monthly during the 3-month follow-up period. Personalized counseling was provided for promoting their diet based on 3-day food records. The following lifestyle intervention goals were established for the participants: daily calorie intake reduced by 500 kcal, receiving 50–60% of daily energy intake from carbohydrates and less than 25% from fat, and fiber intake of at least 12 g/1,000 kcal. Dietary counseling was personalized to each participant according to the assessment results using a software program (MediCheck Careplus, Computer-Aided Nutritional Analysis program of the Korean Association of Health Promotion) based on 3-day food records.

Personalized counseling for promoting exercise behaviors was also provided based on the average frequency of exercise each week. At each visit, the participants received an individual guideline for increasing their level of physical activity according to their results on the “last 7 d recall” domain of the short-form International Physical Activity Questionnaire (IPAQPerforming moderate-intensity exercise for at least 30 min/day on 5 days per week or 150 min/week (e.g., walking, swimming, bicycling, or badminton) was recommended. The type, duration, and frequency of exercise were individualized according to the lifestyle or health condition of each participant.

### Outcomes and assessments

The primary outcome was remission of NAFLD including fatty liver as determined by an improvement of one or more grade of fatty liver on abdominal US and one or more grade of liver stiffness (LS) as measured by MRE at month 12. The secondary outcomes were the changes in metabolic factors and M2BPGi according to anthropometric and biochemical measurements at month 12.

1. Assessment of changes in metabolic factors and M2BPGi

At baseline and at months 3, 6, 9, and 12, anthropometric and biochemical measurements were made to assess liver function and blood lipid, fasting blood sugar (FBS), insulin, and M2BPGi levels. The biochemical parameters were measured using the Hitachi 7600 analyzer (Hitachi, Tokyo, Japan). Serum M2BPGi levels were measured using a chemiluminescence enzyme immunoassay (HISCL-5000, Sysmex, Kobe, Japan).

2. Fatty liver assessment using US and MRI-PDFF

Abdominal US was performed to assess hepatic steatosis at baseline and month 12. The presence and degree of fatty liver were defined according to the obtained results. Abdominal US was performed while applying standard criteria for diagnosing fatty liver based on parenchymal brightness, liver-to-kidney contrast, deep-beam attenuation, and bright vessel walls. Severity is often graded clinically using the following four-point scale: (1) normal (grade 0, absence of steatosis with normal liver echogenicity); (2) mild (grade 1, mild steatosis, the liver had a higher echogenicity than the right renal cortex, however, the echogenic wall of the main portal vein was preserved); (3) moderate (grade 2, moderate steatosis, impaired echogenicity of the main portal vein wall); and (4) severe (grade 3, severe steatosis, impaired visualization of the posterior hepatic parenchyma or the diaphragm) [17]. MRI-PDFF was performed to assess liver steatosis at baseline and month 12 on fifty-five participants at three centers that have software program for PDFF. Twenty-four regions of interest (ROIs) were established for measuring PDFF. The mean and standard-deviation values of fat levels at each segment, lobe, and whole liver were determined. The weighted mean for each segment was calculated with regard to their segmental volumes, and with reference to a previous study that measured intrahepatic segmental volumes [18].

3. LS measurement using MRE

MRE was performed to measure LS at baseline and month 12 on fifty-five participants at three centers that have MRE. MRE was performed using either MRE hardware (GE Healthcare, Waukesha, WI, USA) with a 1.5-T imaging system or a 1.5-T whole-body magnetic resonance unit (Gyroscan Intera, Philips Medical Systems, Best, the Netherlands) with a four-element torso coil. The two-dimensional MRE protocols used were similar to those described previously [20, 21]. The LS values of hepatic parenchyma were measured using MRE by drawing four ROIs on the elastogram. The cutoff values for liver fibrosis in the present study were 1.94, 2.90, and 3.6 kPa for fibrosis stages F1 (mild fibrosis), F2 (significant fibrosis), and ≥F3 (advanced fibrosis), respectively, according to MRE [19–21].

### Statistical analysis

All statistical analyses were performed using SAS software (version 9.4, SAS Institute, Cary, NC, USA). The sample size was estimated as follows: NAFLD remission rates (the primary outcome) were considered for computing the sample size. An observation study found that 16% of patients exhibited spontaneous NAFLD remission within 1–2 years [22]. Assuming that 32% of patients in the present study would have NAFLD remission with 90% statistical power (α = 0.05, 1–β = 0.9) in detecting the difference at a 5% significance level using the chi-square test, 30% of the participants would be lost to follow-up, and so a total sample of 116 participants was required.

Data were expressed as mean±standard-deviation or frequency (percentage) values. Differences in the clinical characteristics of the participants at baseline and at the time of assessment during the 12-month intervention period were analyzed using repeated-measures ANOVAs and chi-square tests. Post-hoc comparisons were performed using Bonferroni adjustment for multiple comparisons. Differences in measurements between baseline and month 12 were determined using Cochran’s Q test. Comparisons between the remission and non-remission groups at month 12 were performed using paired *t*-tests. Multivariate logistic regression analyses were performed to identify the factors associated with NAFLD remission at month 12. Pearson’s correlation analysis was used to determine the relationship between PDFF and M2BPGi levels. A probability value of *P*<0.05 was considered significant.

## Results

**Fig 1:**
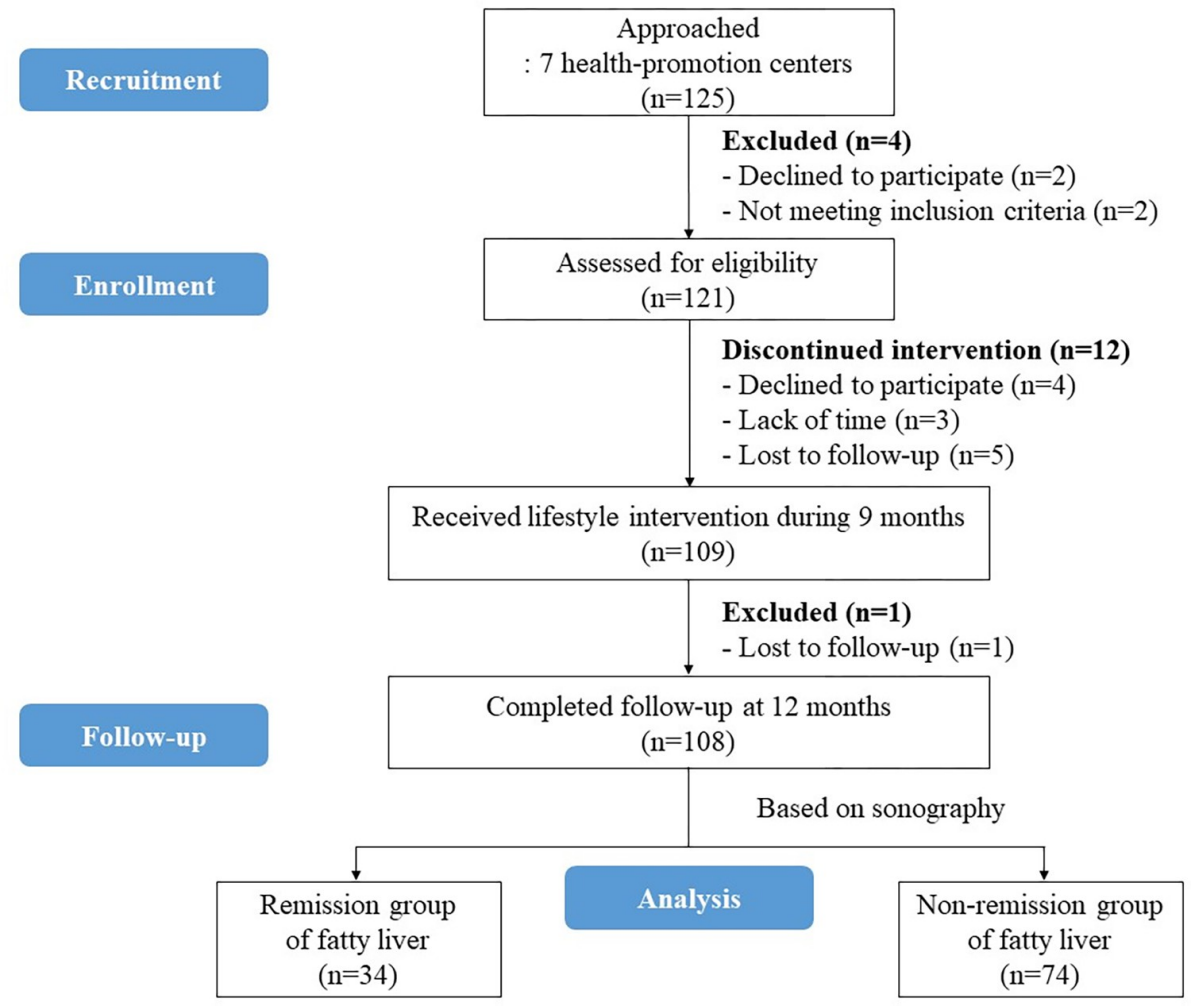
CONSORT flow diagram.

The 121 individuals were initially recruited from health examinees at 7 health-promotion centers. The 109 eligible participants received lifestyle interventions between July 2, 2021 and July 30, 2022. One participant ceased participation after 6 months, and so 108 were finally analyzed after the 12-month intervention (Fig 1).

### Baseline characteristics

The baseline characteristics of the study population are listed in Table 1. The participants were aged 51.1±12.4 years (mean±standard deviation), with a male predominance (57.4%). The BMI was 28.9±3.3 kg/m^2^ and waist circumference (WC) was 94.6±8.2 cm. The proportions of participants with mild, moderate, and severe fatty liver grades measured on US were 32.4%, 61.1%, and 6.5%, respectively. The proportions of those with fibrosis at stages F0, F1, F2, and F3 measured on MRE were 30.9%, 60.0%, 7.3%, and 1.8%, respectively.

**Table 1:** Clinical characteristics of the participants at baseline and different follow-up times.

		Baseline (T0)		After 3 months (T3)		After 6 months (T6)		After 9 months (T9)		After 12 months (T12)		*P*	Post hoc^§^
BMI, kg/m^2^		28.90 ± 3.29		28.52 ± 3.23		28.51 ± 3.24		28.52 ± 3.25		28.36 ± 3.23		<0.001	T0>T3, T9>T12
WC, cm		94.55 ± 8.16		93.33 ± 7.85		93.18 ± 8.47		92.42 ± 8.09		93.13 ± 8.52		<0.001	T0>T3, T6>T9
SBP, mmHg		124.92 ± 12.96		122.48 ± 11.54		122.92 ± 11.21		123.97 ± 12.93		121.54 ± 11.76		0.023	T0>T3, T9>T12
DBP, mmHg		78.33 ± 10.00		77.68 ± 8.72		78.36 ± 8.76		77.86 ± 9.76		76.44 ± 9.12		0.136	
AST, IU/L		32.86 ± 14.55		32.66 ± 20.39		30.95 ± 14.43		32.19 ± 18.27		32.28 ± 20.43		0.865	
ALT, IU/L		39.80 ± 21.96		37.58 ± 28.31		38.19 ± 28.13		37.44 ± 28.75		36.83 ± 29.76		0.864	
r-GTP, IU/L		62.51 ± 122.04		51.31 ± 70.05		52.93 ± 87.28		56.79 ± 88.53		54.84 ± 97.38		0.060	
Albumin, g/dL		4.52 ± 0.24		4.51 ± 0.25		4.51 ± 0.25		4.51 ± 0.27		4.49 ± 0.24		0.506	
hs-CRP, mg/dL		0.22 ± 0.22		0.20 ± 0.26		0.21 ± 0.26		0.19 ± 0.15		0.18 ± 0.16		0.563	
TC, mg/dL		199.13 ± 40.44		194.37 ± 38.55		195.51 ± 42.94		189.98 ± 38.10		190.52 ± 40.18		0.044	
TG, mg/dL		199.69 ± 208.59		167.33 ± 100.21		169.22 ± 126.84		168.09 ± 119.68		170.51 ± 103.51		0.033	T0>T3
HDL-C, mg/dL		47.57 ± 11.44		48.66 ± 11.92		49.84 ± 13.60		49.32 ± 13.01		49.09 ± 12.85		0.006	T3<T6
LDL-C, mg/dL		113.94 ± 37.06		112.50 ± 34.72		111.79 ± 42.48		107.05 ± 38.43		107.30 ± 37.35		0.086	
FBS, mg/dL		114.21 ± 34.85		107.76 ± 26.63		111.55 ± 28.33		111.90 ± 28.33		110.83 ± 24.80		0.011	T0>T3, T3<T6
HbA1c, %		6.20 ± 1.18		5.98 ± 0.83		6.02 ± 0.87		6.07 ± 0.89		6.06 ± 0.82		0.002	T0>T3
Insulin, mIU/L		12.88 ± 6.75		14.26 ± 15.93		13.89 ± 12.18		12.99 ± 10.55		11.88 ± 7.07		0.230	
HOMA-IR		3.66 ± 2.30		3.85 ± 4.42		4 ± 4.6		3.76 ± 3.74		3.33 ± 2.35		0.362	
Fat fraction, %^†^		17.30 ± 8.07								14.65 ± 8.69		0.040	T0>T12
FL grade based on US													
	Normal	0								10	(9.3)	<0.001	
	Mild	35	(32.4)							43	(39.8)		
	Moderate or severe	73	(67.6)							55	(50.9)		
FL remission of one or more grade on US^‡^										34	(31.5)	-	
M2BPGi, COI		0.69 ± 0.28		0.58 ± 0.25		0.63 ± 0.28		0.69 ± 0.32		0.67 ± 0.26		<0.001	T0>T3, T3<T6, T6<T9
FIB-4		1.12 ± 0.5		1.12 ± 0.54		1.12 ± 0.53		1.16 ± 0.57		1.19 ± 0.6		0.062	
APRI		0.33±0.17		0.32 ± 0.2		0.31 ± 0.16		0.32 ± 0.23		0.34 ± 0.29		0.611	
NFS		-2.21±1.24		-2.3 ± 1.28		-2.25 ± 1.32		-2.19 ± 1.29		-2.1 ± 1.27		0.035	T3<T12, T6<T12
LS based on MRE													
	F0	17	(30.9)							14	(25.5)	0.507	
	F1	33	(60)							39	(70.9)		
	≥F2	5	(9.1)							2	(3.6)		
	Means ± SD, kPa^†^	2.13 ± 0.61								2.23 ± 0.57		0.099	
LS remission of one or more grade on MRE^‡^										12	(21.8)	-	

Data are mean±standard deviation or *n* (%) values

Abbreviation: BMI, body mass index; WC, waist circumference; SBP, systolic blood pressure; DBP, diastolic blood pressure; AST, aminotransferase; ALT, alanine aminotransferase; r-GTP, gamma-glutamyl transpeptidase; hs-CRP, high-sensitivity C-reactive protein; TC, total cholesterol; TG, triglyceride; HDL-C, HDL-cholesterol; LDL-C, LDL-cholesterol; FBS, fasting blood sugar; HbA1C, hemoglobin A1_C_; FF, fat fraction; US, ultrasonography; FL, fatty liver; M2BPGi, Mac-2 binding protein glycosylation isomer; COI, cutoff index; APRI, aspartate transaminase -to-platelet ratio index; FIB-4, Fibrosis-4 index; NFS, NAFLD fibrosis score; LS, liver stiffness; MRE, magnetic resonance elastography; kPa, kilopascal.

^†^ There are missing data

^‡^ Liver status improvement (fatty liver or liver fibrosis)

^§^ Post-hoc comparisons were performed using Bonferroni adjustment for multiple comparisons

### Primary outcome

The primary outcome of NAFLD remission (fatty liver as determined by an improvement of one or more grade of fatty liver on abdominal US) occurred in 34 (31.5%) of the 108 participants after the 12-month lifestyle intervention (*P* = 0.002). Twelve participants (21.8%) of the 55 participants who underwent MRE had improvement of liver stiffness (LS) based on MRE in the present study. The participant numbers of fatty liver worsening (in one case) or improving after 12 months is shown in Table A in S2 Table. The hepatic fat fraction (FF) also decreased from 17.30±8.07% to 14.65±8.69% (*P* = 0.04). However, LS was not improved at month 12 (Table 1). In the comparison of the remission and non-remission groups, hepatic FF had significantly changed in the remission group between baseline and month 12 (*P*<0.001) (Table 2). BMI, WC, serum alanine aminotransferase (ALT), and HDL-C were associated with NAFLD remission in the univariate analyses (all *P*<0.05). After adjusting for age, sex, and all variables at baseline and month 12 in the multivariate analysis, WC (OR = 0.82, 95% CI = 0.67–1.00, *P* = 0.05) and HDL-C (OR = 1.17, 95% CI = 1.03–1.32, *P* = 0.014) were associated with remission (Table 3).

**Table 2:** Comparison between the remission and non-remission groups of NAFLD from baseline to month 12.

		Baseline	Month 12	Difference between baseline and month 12	*P*
BMI, kg/m^2^					
	Remission group	29.29 ± 3.30	28.04 ± 3.30	−1.25	<0.001
	Non-remission group	28.72 ± 3.30	28.50 ± 3.20	−0.22	
WC, cm					
	Remission group	96.05 ± 7.99	92.13 ± 8.81	−3.92	<0.001
	Non-remission group	93.86 ± 8.20	93.59 ± 8.41	−0.27	
SBP, mmHg					
	Remission group	123.74 ± 11.05	121.41 ± 11.06	−2.33	0.539
	Non-remission group	125.46 ± 13.62	121.59 ± 12.14	−3.87	
DBP, mmHg					
	Remission group	76.82 ± 9.51	75.44 ± 9.90	−1.38	0.691
	Non-remission group	79.03 ± 10.21	76.89 ± 8.76	−2.14	
AST, IU/L					
	Remission group	34.76 ± 16.21	30.09 ± 17.08	−4.67	0.110
	Non-remission group	31.99 ± 13.75	33.28 ± 21.83	1.29	
ALT, IU/L					
	Remission group	41.88 ± 24.13	30.88 ± 26.91	−11	0.020
	Non-remission group	38.84 ± 20.99	39.57 ± 30.77	0.73	
r-GTP, IU/L					
	Remission group	83.68 ± 198.8	67.32 ± 160.25	−16.36	0.172
	Non-remission group	52.78 ± 60.49	49.11 ± 46.73	−3.67	
Albumin, g/dL					
	Remission group	4.45 ± 0.25	4.49 ± 0.21	0.04	0.037
	Non-remission group	4.56 ± 0.24	4.49 ± 0.25	−0.07	
TC, mg/dL					
	Remission group	197.50 ± 36.55	188.85 ± 43.73	−8.65	0.995
	Non-remission group	199.88 ± 42.32	191.28 ± 38.74	−8.6	
TG, mg/dL					
	Remission group	219.44 ± 211.67	156.53 ± 88.29	−62.91	0.198
	Non-remission group	190.62 ± 207.98	176.93 ± 109.76	−13.69	
HDL-C, mg/dL					
	Remission group	46.56 ± 12.80	51.29 ± 16.14	4.73	0.001
	Non-remission group	48.04 ± 10.82	48.08 ± 11.00	0.04	
FBS, mg/dL					
	Remission group	117.09 ± 46.73	111.5 ± 30.25	−5.59	0.524
	Non-remission group	112.89 ± 28.06	110.53 ± 22.07	−2.36	
HbA1c, %					
	Remission group	6.30 ± 1.17	6.10 ± 0.91	−0.2	0.434
	Non-remission group	6.15 ± 1.19	6.05 ± 0.78	−0.1	
Insulin, mIU/L					
	Remission group	13.74 ± 7.07	10.94 ± 5.84	−2.8	0.035
	Non-remission group	12.48 ± 6.61	12.32 ± 7.56	−0.16	
FF, %†					
	Remission group	17.51 ± 9.34	6.39 ± 3.87	−11.12	<0.001
	Non-remission group	17.25 ± 7.91	16.71 ± 8.34	−0.54	
M2BPGi, COI					
	Remission group	0.73 ± 0.22	0.67 ± 0.25	−0.06	0.330
	Non-remission group	0.68 ± 0.30	0.67 ± 0.27	−0.01	
Liver fibrosis based on MRE					
	Remission group	2.08 ± 0.68	2.06 ± 0.68	−0.02	0.178
	Non-remission group	2.15 ± 0.58	2.30 ± 0.52	0.15	

Data are mean±standard-deviation values

Abbreviation: NAFLD, non-alcoholic fatty liver disease; BMI, body mass index; WC, waist circumference; SBP, systolic blood pressure; DBP, diastolic blood pressure; AST, aminotransferase; ALT, alanine aminotransferase; r-GTP, gamma-glutamyl transpeptidase; TC, total cholesterol; TG, triglyceride; HDL-C, HDL-cholesterol; FBS, fasting blood sugar; HbA1_c_, hemoglobin A1C; FF, fat fraction; M2BPGi, Mac-2 binding protein glycosylation isomer; COI, cutoff index; MRE, magnetic resonance elastography.

**Table 3:** Associated metabolic factors with remission of NAFLD based on abdominal ultrasonography at month 12.

	Model 1			Model 2		
	OR	95% CI	*P*	OR	95% CI	*P*
BMI, kg/m^2^	0.49	0.33, 0.74	0.001	0.63	0.31, 1.26	0.193
WC, cm	0.81	0.72, 0.92	0.001	0.82	0.67, 1.00	0.050
AST, IU/L	0.98	0.95, 1.01	0.175	0.96	0.89, 1.05	0.373
ALT, IU/L	0.98	0.95, 1.00	0.041	1.02	0.96, 1.08	0.522
r-GTP, IU/L	0.99	0.98, 1.01	0.386	1.00	0.97, 1.04	0.885
Albumin, g/dL	5.10	0.48, 54.68	0.178	0.93	0.04, 24.25	0.967
TC, mg/dL	1.00	0.99, 1.01	0.909	1.00	0.98, 1.02	0.725
TG, mg/dL	1.00	0.99, 1.00	0.188	1.00	1.00, 1.01	0.351
HDL, mg/dL	1.12	1.04, 1.20	0.002	1.17	1.03, 1.32	0.014
HbA1c, %	0.88	0.40, 1.94	0.757	0.87	0.18, 4.09	0.858
Insulin, mIU/L	0.92	0.84, 1.01	0.079	0.98	0.87, 1.12	0.791

Note: model 1 was adjusted for age, sex, and baseline values of each variable

model 2 was adjusted for age, sex, and all variables at baseline and month 12

Abbreviation: NAFLD, non-alcoholic fatty liver disease; BMI, body mass index; WC, waist circumference; AST, aminotransferase; ALT, alanine aminotransferase; r-GTP, gamma-glutamyl transpeptidase; TC, total cholesterol; TG, triglyceride; HDL-C, HDL-cholesterol; HbA1_C_, hemoglobin A1_C_.

### Secondary outcomes

The lifestyle behavior characteristics of participants were changed from baseline to follow-up times. Exercise level had improved at the 12-month examination (Table B in S2 Table).

**Fig 2:**
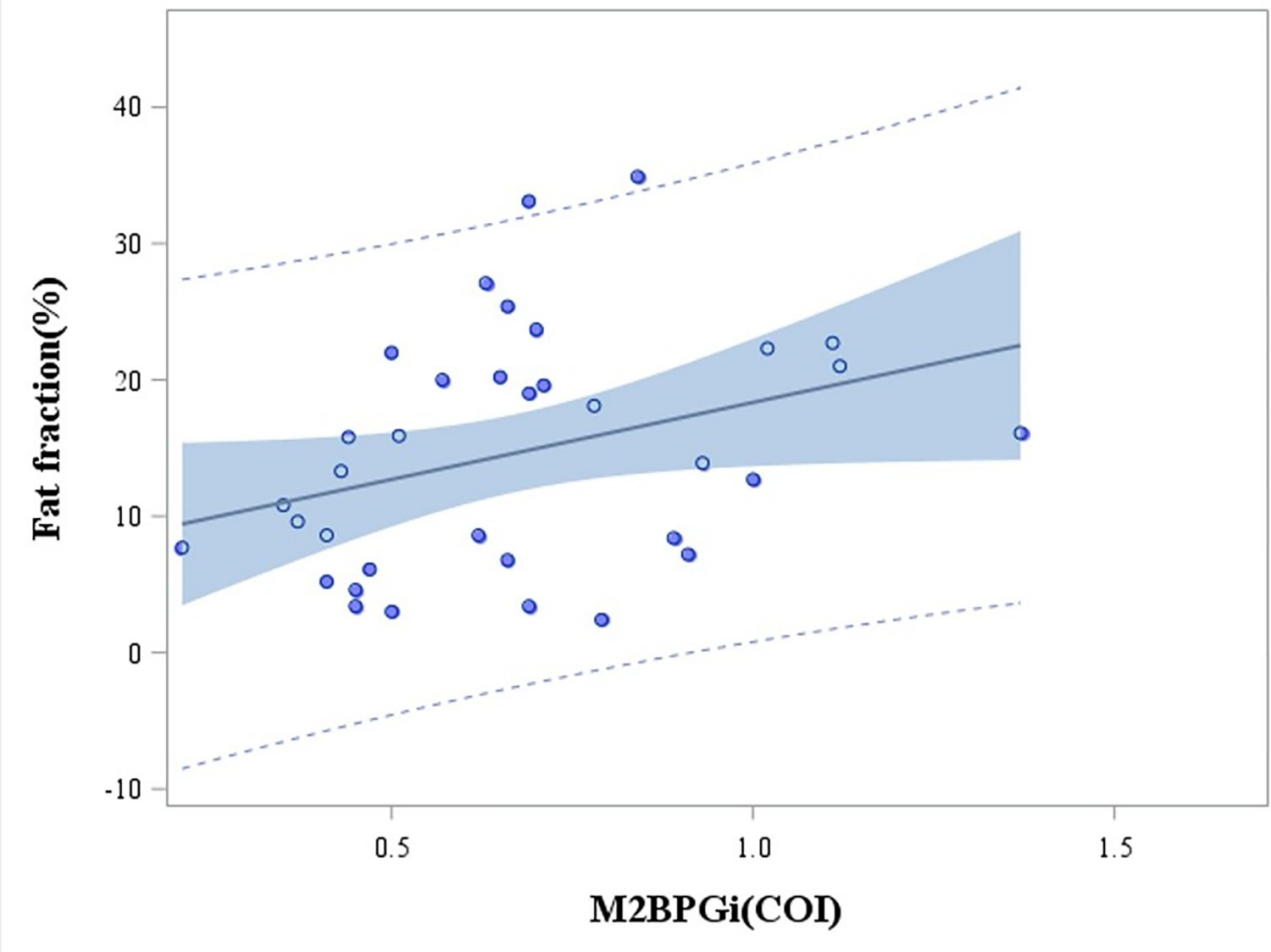
Correlation between fat fraction and M2BPGi.

**Fig 3:**
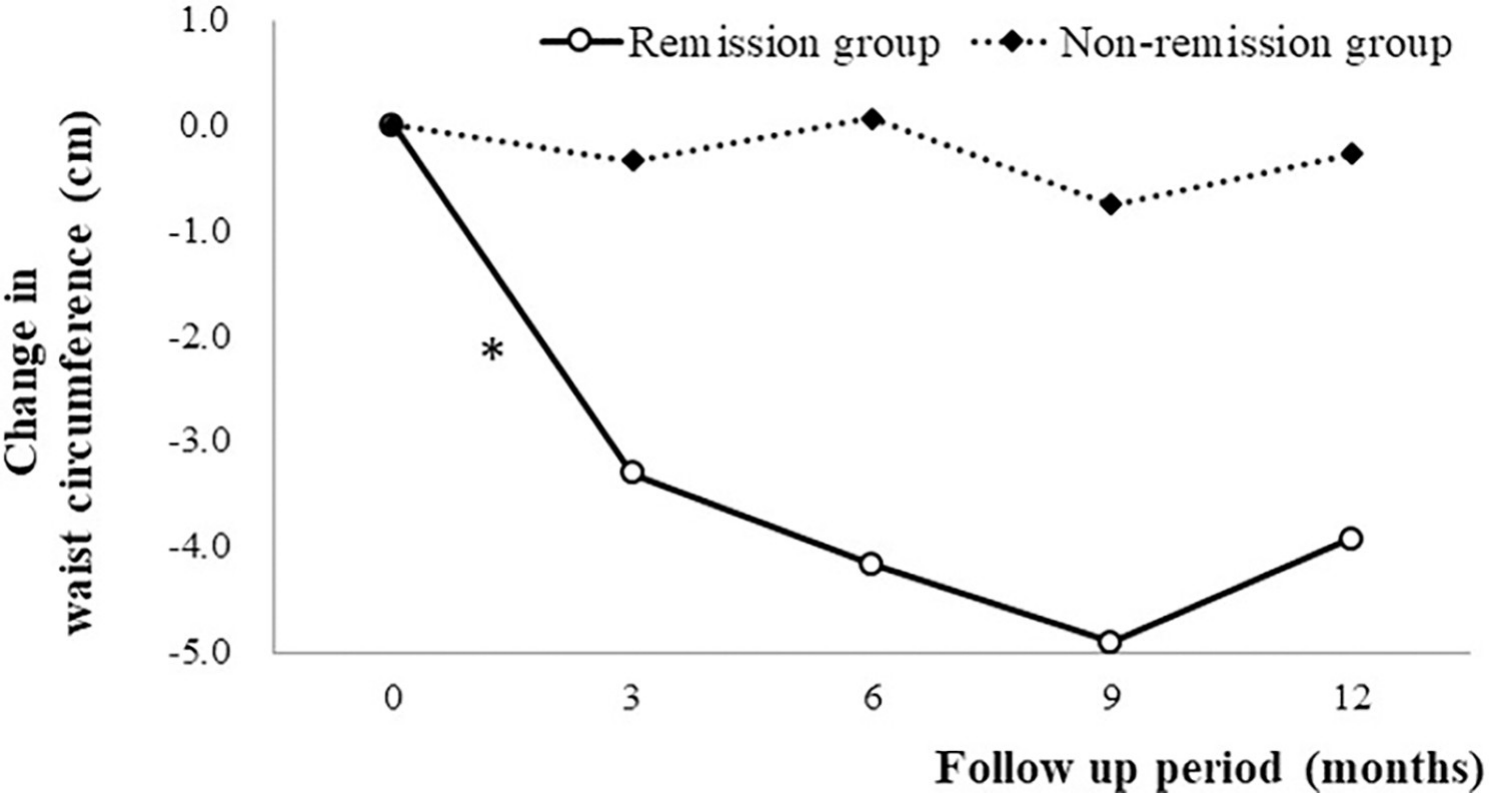
Mean changes in waist circumference from baseline to month 12 in the remission and non-remission groups.

BMI, WC, and systolic blood pressure were decreased at month 12. Blood lipids (total cholesterol [TC], triglycerides [TG], and HDL-C) and FBS were improved (all *P*<0.05). Moreover, M2BPGi was also decreased (*P*<0.001) (Table 1) and was moderately correlated with hepatic FF (*r* = 0.33, *P* = 0.05) (Fig 2). In the comparison between the remission and non-remission groups, BMI, WC, ALT, albumin, HDL-C, and insulin were significantly changed in the remission group between baseline and month 12 (all *P*<0.05) (Table 2) (Fig 3).

## Discussion

In this single-arm lifestyle intervention study, we found that fatty liver remission occurred in 34 (31.5%) of the 108 participants after the 12-month lifestyle intervention. WC and HDL-C were associated with fatty liver remission. However, LS was not improved at month 12. Metabolic factors including BMI, WC, blood pressure, blood lipids (TC, TG, and HDL-C), FBS, and M2BPGi were improved relative to baseline. M2BPGi was moderately correlated with hepatic FF.

Lifestyle modification using hypocaloric diet and exercise is considered as a first-line intervention for NAFLD treatment and acts through significant weight loss, which is associated with a reduction in liver fat [8, 23]. Several systematic reviews and meta-analyses have found that behavioral modification resulted in reduced hepatic steatosis and NAFLD progression [24–26]. These studies highlighted that more-intensive interventions induced greater weight loss, which can lead to improvements in NAFLD-related liver biomarkers. Our study found at significant difference in WC between baseline and month 3 in the remission group, which was maintained to month 9. WC was a factor associated with NAFLD remission whereas BMI was not after adjusting for age, sex, and all variables at baseline and month 12 in our study. This suggests that NAFLD remission is more closely associated with reduced WC than with BMI. A meta-analysis found that individuals with abdominal obesity as measured by WC had a higher risk of NAFLD than did those with general obesity measured by BMI [27]. Another study also identified that WC was the most-significant risk factor for incident NAFLD among various body-composition variables in South Korean adults [28]. Visceral fat in abdominal obesity was independently associated with hepatic inflammation and fibrosis regardless of insulin resistance. Visceral adipose tissue would not only promote macrophage infiltration and pro-inflammatory cytokine secretion, but also decrease adiponectin secretion which reduces insulin resistance and inflammation. This adipose tissue would result in fatty acids flowing into the liver, and decreases in adiponectin secretion might lead to ectopic fat accumulation in the liver [29–31].

On the other hand, LS was not improved after our 12-month lifestyle intervention. Previous studies found that although weight loss can significantly impact all aspects of NAFLD histology, a goal of 10% weight loss should be considered for inducing fibrosis regression [24–26]. Participants with fatty liver remission in our study achieved small decreases in BMI. Moreover, the participants in our study spanned the spectrum of relatively mild disease as they were recruited from primary healthcare centers. Five (9.1%) participants had fibrosis of stage F2 or worse at baseline, which may not have been sufficient to analyze the impact on LS.

Metabolic factors including blood pressure, blood lipids (TC, TG, and HDL-C), and FBS were improved through the lifestyle intervention without more than 5% weight loss in the present study. Body weight reduction of 5% or more was achieved in seven participants among our study subjects. A study found that the dietary composition in lifestyle interventions can influence the hepatic phenotype and particularly fat content even in the absence of weight loss. These could be assumed based on the available surrogate markers [32]. The lifestyle interventions in our study were applied to the participants based on personalized prescriptions that considered their body weight, diet, and exercise behaviors. The metabolic conditions that coexist with NAFLD are obesity, diabetes, hyperlipidemia, hypertriglyceridemia, hypertension, and metabolic syndrome [1]. Healthy eating and exercise are considered to be other aspects of lifestyle interventions that can exert positive metabolic effects without necessarily involving weight loss [33, 34]. Moreover, multivariate analysis indicated that factors related to the resolution of NAFLD included not only waist circumference but also HDL-C in this study. The dyslipidemia of NAFLD and NASH has previously been characterized by hypertriglyceridemia, elevated LDL-C and total cholesterol and low HDL-C. In the present study, the resolution of NAFLD was associated with significant improvement in HDL-C. HDL-C particles remove excess cholesterol from peripheral tissues including the endothelium via the reverse cholesterol transport pathway. Additionally, HDL-C has anti-inflammatory properties that decrease atherosclerotic plaque development and instability.

M2BPGi was found to have decreased during the 12-month intervention of the present study, and was moderately correlated with hepatic FF. M2BPGi is secreted from hepatic stellate cells and induces Mac-2 expression in Kupffer cells. It activates hepatic stellate cells and increases alpha-smooth-muscle actin expression, which is correlated with liver fibrosis [35]. The serum M2BPGi level has been found to increase with liver fibrosis severity in patients with chronic hepatitis and with NAFLD [36]. Moreover, the M2BPGi level reflects both liver fibrosis and function [37]. Few studies have independently identified the utility of M2BPGi in the degree of fatty liver remission, and so further investigation is required.

This study has some limitations. First, NASH, estimated using MRE, was only present in a small proportion of our participants, which meant that we could not assess the effect of lifestyle intervention on liver fibrosis remission. This was due to the participants being recruited from the general population. Second, our study was a single-arm trial. Third, liver biopsy was not performed and not directly compared with the histologic grade, although a biopsy is the most accurate NAFLD diagnosis method. However, MRE is a noninvasive method that allowed accurate assessments of NASH and advanced liver fibrosis for many subjects within the community in which liver biopsies were less feasible. Fourth, this study had used the US as the diagnosis of NAFLD and assessment of fatty liver remission which showed limited sensitivity and specificity. However, the US is the cheaper method and has been the most common modality used in clinical practice. Moreover, abdominal US may have the intra- and inter-observer variability. However, this study used the same measurement protocol for fatty liver assessment to reduce intra- and inter-observer variability, although abdominal US of all the participants did not be performed by same physician in this study.

## Conclusions

Lifestyle intervention was effective in achieving liver steatosis remission and cardiovascular and metabolic health. However, changing the lifestyle behaviors of our participants would not be sufficient for liver fibrosis remission. Further investigation is required to determine the optimal lifestyle intervention method that would improve liver fibrosis for application in patients with NAFLD in primary healthcare.

## Supporting information

S1 File Study protocol. (PDF)

S2 File Study protocol (Korean). (PDF)

S1 Table TREND checklist. (PDF)

S2 Table Changes at follow-up times. (PDF)
