# Supplementary material for: Changes in nonalcoholic fatty liver disease and M2BPGi due to lifestyle intervention in primary healthcare
Source: PLoS One. 2024 Feb 29;19(2):e0298151. doi: 10.1371/journal.pone.0298151 (PMC10903829; doi:10.1371/journal.pone.0298151)
Supplement: S1 File — (PDF) [file pone.0298151.s001.pdf]

Study Title

Changes in nonalcoholic fatty liver disease and M2BPGi due to lifestyle intervention in primary healthcare

Short title: Changes in NAFLD and M2BPGi due to lifestyle intervention

1. GENERAL INFORMATION

1.1 Protocol Title

Changes in nonalcoholic fatty liver disease and M2BPGi due to lifestyle intervention in primary healthcare

1.2 Conflict of interest

No special funding was received for this study, and no conflicts of interest exist in the planning, conduct, or reporting of the study that would affect the results of the study or the interpretation of the results.

1.3 Investigators Responsible for Conducting the Research

Korea Association of Health Promotion (KAHP)

1.4 Trial Registration

This clinical trial has been registered with cris.nih.go.kr (KCT0006380)

2. SYNOPSIS

TITLE

Changes in nonalcoholic fatty liver disease and M2BPGi due to lifestyle intervention in primary healthcare

OBJECTIVES

The aim of this research is to determine the efficacy of personalized lifestyle interventions on NAFLD remission.

PRIMARY HYPOTHESIS

NAFLD development is related to lifestyle factors such as a high-calorie diet with reduced physical activity and exercise. Effectively treating NASH is required to interrupt the disease progression. Lifestyle changes that focus on weight loss remain the cornerstone of NASH treatment. Lifestyle interventions combined with a 10% decrease in body weight may improve the states of steatosis, inflammation, and even fibrosis.

DESIGN

This is a 12-month single arm within-participant intervention study involving a 9-month intervention period and a 3-month follow-up.

BLINDING/MASKING

Since our intervention involves the management of dietary and exercise is integral for lifestyle intervention, it is not possible or recommended to blind participants in the intervention. The dietitian delivering the intervention will be blinded to complete data collection and analysis. As such, the outcome assessor will also be blinded to the intervention.

OUTCOMES

The primary outcome is NAFLD remission at month 12 as measured on US and magnetic resonance elastography.

STUDY DURATION

12 months

INTERVENTION/S

The following lifestyle intervention goals will be established for the participants: daily calorie intake reduced by 500 kcal, receiving 50–60% of daily energy intake from carbohydrates and less than 25% from fat, and fiber intake of at least 12 g/1,000 kcal. Dietary counseling will be personalized to each participant according to the assessment results using a software program (MediCheck Careplus, Computer-Aided Nutritional Analysis program of the Korean Association of Health Promotion) based on 3-day food records. Personalized counseling for promoting exercise behaviors will be also provided based on the average frequency of exercise each week.

NUMBER OF PARTICIPANTS

116

POPULATION

Adults (20-80 yrs) with NAFLD

SELECTION AND ENROLMENT

Inclusion criteria

• Adults aged 20-80 yrs

• Residing within South Korea

•Health examinees who underwent health checkups at seven health-promotion centers

• body mass index (BMI) of ≥25 kg/m^2

* fatty liver of at least mild severity as measured by abdominal US

Exclusion criteria

* history of viral hepatitis or hepatocellular malignancy

* secondary causes of fatty liver

* high alcohol consumption (>210 g for males and > 140 g for females weekly)

3. RATIONALE / BACKGROUND

The increasing prevalence of obesity and diabetes is resulting in nonalcoholic fatty liver disease (NAFLD) also becoming increasingly prevalent. Approximately 25% of the worldwide population was estimated to have NAFLD [1], affecting 60% of patients with diabetes [2] and 90% of people with obesity [3]. The spectrum of NAFLD ranges from simple steatosis to nonalcoholic steatohepatitis (NASH). Patients with NAFLD, and especially those with NASH, may eventually progress to fibrosis, leading to cirrhosis and hepatocellular carcinoma. The fibrosis stage of liver disease is known to be associated with the long-term outcomes in individuals with NAFLD [4, 5].

NAFLD development is related to lifestyle factors such as a high-calorie diet with reduced physical activity and exercise. Effectively treating NASH is required to interrupt the disease progression [6]. Lifestyle changes that focus on weight loss remain the cornerstone of NASH treatment [7]. Lifestyle interventions combined with a 10% decrease in body weight may improve the states of steatosis, inflammation, and even fibrosis [8]. Recent studies were conducted at a tertiary medical center and expert centers [8, 9]. Some studies used interventions that involved tightly controlled diets over short periods ranging several weeks to months [10–12]. However, it is unlikely that participants can adhere to such diets for a long time to achieve NAFLD remission, and hence a community-based lifestyle modification program that could be

applied in primary healthcare centers is needed.

Imaging technologies such as ultrasonography (US), magnetic resonance imaging (MRI), and transient elastography have also been widely used for the assessment of liver steatosis and stiffness. Magnetic resonance elastography (MRE) has been demonstrated to show high diagnostic accuracy for liver fibrosis [13, 14]. An MRI-based technique of measuring the proton-density fat fraction (PDFF) was developed to measure the hepatic fat level. This technique can accurately measure the fat levels of all hepatic areas [15, 16]. However, considering its cost and need for MRI equipment, it is inappropriate to apply these techniques to periodic follow-ups of the degree of hepatic fat and fibrosis in individuals with NAFLD in primary healthcare.

4. AIMS / OBJECTIVES / HYPOTHESES

This study aims to determine the efficacy of personalized lifestyle interventions on NAFLD remission, and on the improvement of metabolic factors and Mac-2-binding protein glycosylated isomer (M2BPGi) in primary healthcare.

5. PARTICIPATING SITES

This project will include both telephone and in-person contact. Telephone intervention will be conducted via cellphone. Clinic visits will be conducted from a private clinic room at each Health promotion center.

6. RESEARCH PLAN / STUDY DESIGN

6.1 Type of study

A 12-month single arm, within-participant intervention study.

6.2 Population / sample size

NAFLD remission rates (the primary outcome) will be considered for computing the sample size. An observation study found that 16% of patients exhibited spontaneous NAFLD remission within 1–2 years [17]. Assuming that 32% of patients in the present study would have NAFLD

remission with 90% statistical power (α=0.05, 1–β=0.9) in detecting the difference at a 5% significance level using the chi-square test, 30% of the participants would be lost to follow-up, and so a total sample of 116 participants was required.

6.3 Statistical analyses

All statistical analyses will be performed using SAS software (version 9.4, SAS Institute, Cary, NC, USA). Differences in the clinical characteristics of the participants at baseline and at the time of assessment during the 12-month intervention period will be analyzed using repeated-measures ANOVAs and chi-square tests. Post-hoc comparisons will be performed using Bonferroni adjustment for multiple comparisons. Differences in measurements between baseline and month 12 will be determined using Cochran's Q test. Comparisons between the remission and non-remission groups at month 12 will be performed using paired *t*-tests. Multivariate logistic regression analyses will be performed to identify the factors associated with NAFLD remission at month 12. Pearson's correlation analysis will be used to determine the relationship between PDFF and M2BPGi levels. A probability value of $P<0.05$ will be considered significant.

6.4 Recruitment and selection of participants (including informed consent)

Participants will be recruited among health examinees who underwent health checkups at seven health-promotion centers in five South Korean cities. Individuals wishing to take part in the study will be required to make initial contact with the research team by calling. The survey will involve multiple screening questions to determine whether or not interested individuals are eligible to participate in the study. Survey responses will be assessed in the order they are received by investigators against the pre-specified inclusion/exclusion criteria. Potentially eligible participants will receive a phone call from investigator to clarify responses, provide additional screening information (e.g.completed confirmation) and if deemed eligible, schedule their first clinic visit. At this point, participants will also be asked to provide contact details so they can be informed of their patient's decision to be involved in the study.

6.5 Lifestyle Intervention Period

These lifestyle interventions will be implemented between July 2, 2021 and July 30, 2022. Dietitians at seven health-promotion centers will be instructed about the operating protocol and provided with a manual to ensure that the trial will be standardized. Participants received six sessions (at baseline, and after 1, 2, 3, 6, and 9 months) at visits to the health promotion centers

over 12 months. In addition to sessions through center visits, phone-based intervention and self-monitoring at 4-, 5-, 7-, and 8-month will be provided during the 9-month intervention period

6.5.1 dietary intervention

Personalized counseling will be provided for promoting their diet based on 3-day food records. The following lifestyle intervention goals will be established for the participants: daily calorie intake reduced by 500 kcal, receiving 50–60% of daily energy intake from carbohydrates and less than 25% from fat, and fiber intake of at least 12 g/1,000 kcal. Dietary counseling will be personalized to each participant according to the assessment results using a software program (MediCheck Careplus, Computer-Aided Nutritional Analysis program of the Korean Association of Health Promotion) based on 3-day food records.

6.5.2 exercise intervention

Personalized counseling for promoting exercise behaviors will be also provided based on the average frequency of exercise each week. At each visit, the participants received an individual guideline for increasing their level of physical activity according to their results on the "last 7 d recall" domain of the short-form International Physical Activity Questionnaire (IPAQPerforming moderate-intensity exercise for at least 30 min/day on 5 days per week or 150 min/week (e.g., walking, swimming, bicycling, or badminton) was recommended. The type, duration, and frequency of exercise willbe individualized according to the lifestyle or health condition of each participant.

6.6 Follow Up

The end of the intervention period will mark the end of the active education period. However, participants will be asked to schedule for 3 months follow-up period with dietitians for the purposes of collecting self-monitoring data (Figure 1).

6.7 Schedule of intervention and follow-up

The schedule of events from first contact to follow-up (final contact) is displayed in Figure 1.

6.8 Informed consent process

Potential participants will be independently responsible for contacting the researchers to make initial contact and express their interest to participate in the study (via email or phone to freely

provide contact details). All participants will be required to complete the survey. All potentially eligible participants will be required to provide their informed written consent by completing the survey and ticking a box to confirm they have read the information and fully understand the study details.

7. ETHICAL CONSIDERATIONS

We will obtain ethics approval from the Korea Association of Health Promotion and will comply with all standards set by their human ethics committee.

7.1 Avoiding real or perceived coercion (recruitment)

Our method of recruitment, as outlined in section 6.4, is via phone call, with all consented prospective health examinees reaching out to researchers. Therefore, there is no risk of real or perceived coercion in the recruitment process.

7.2 Privacy & confidentiality in the dissemination of research results

The overall results of this research project will be disseminated via journal publication(s), conference presentations, and educational seminars. Results will be primarily reported as means and variances; individuals will not be identifiable in any dissemination of research results. Where reference to individual data is appropriate/necessary, this will be reported as "one participant", etc. rather than using participant ID codes or any potentially identifying information.

7.3 Potential risks involved with participation in research

7.3.1 Discomfort or infection

There is a small risk of discomfort infection associated with venipuncture blood draws. In all cases, standard antiseptic protocols and procedures will be used to minimize the risk of physical harm. It should be noted that the expected physical harm from participating in this research is typical of health checkup.

7.3.2 Social distress

There is also a risk of social distress with increased travel and time commitments associated with participation in the study given participants will need to attend several study visits, diet

sessions and health promotion centers. To reduce the burden on participants, sessions will be scheduled at a time of day for the participants.

8. SAFETY CONSIDERATIONS

8.1 Adverse event definitions

For the purposes of this study, we will define adverse events as any incident that results in the participant requiring health care of which is directly attributed to involvement in the study. The expected risk of serious adverse events experienced as a result of the dietary intervention is low. However, participants will be advised to report details of any adverse events directly to the study dietitian or physician at any point during the intervention periods.

9. OUTCOMES

All primary and secondary outcomes will be measured as refer to Figure 1. Refer to Figure 1 for a clear description of what outcomes are being measured at what time-points throughout the duration of the study for each participant. Refer to Figure 1 for an overview of this information.

9.1 Primary outcome

- remission of NAFLD including fatty liver as determined by an improvement of at least one grade of fatty liver on abdominal US and liver stiffness (LS) as measured by MRE at month 12

9.2 Secondary outcome

- the changes in metabolic factors and M2BPGi according to anthropometric and biochemical measurements at month 12.
  - Body Mass Index (BMI) (kg/m^2) [height and weight measured using standard procedures by study dietitian^]
  - Waist circumference (cm) [measured at the midline point between the lowest rib and iliac crest by study dietitian^]
  - Resting blood pressure (BP) (mmHg) [measured after 5 minutes of seated rest by study

dietitian^]

• biochemical measurements

; liver function and blood lipid, fasting blood sugar (FBS), and insulin levels

10. DATA MANAGEMENT

10.1 Data collection

We will collect data from the following sources:

- Survey (including consent form)
- Pathology and diagnostic radiology results provided by core laboratory and radiologic examination centers at KAHP
- Outcome measurements taken at study visits with the study dietitian (e.g., weight, waist circumference, blood pressure, questionnaires)
- Self-monitoring data provided by participants (e.g., body weight, dietary intake data)
- Any other relevant information provided by participants at any time-point throughout the study (e.g., details of adverse events, etc.)

10.2 Data storage

As mentioned in section 10.1 all participant data collected during this study will be stored securely on KAHP data base.

10.3 Data usage

Relevant data items will be extracted or exported from KAHP data base in re-identifiable format (using study IDs only) and inputted into Microsoft Excel where all relevant data collected for each participant throughout the duration of the study will be merged for analysis. Data will be manipulated/analyzed by study investigators on password-protected laptops or computers during the project.

11. TIMELINES / MILESTONES

We anticipate the project to start on June 3, 2021 and end on July 30, 2022 (13 months).

The recruitment period will start on 3rd June 2021 and end on the 28th June 2021 (~1 month) or when 116 participants have been successfully recruited. Participants will enter the study at different time points, depending on when they are recruited. Including the follow-up period, the total time each participant will be in the study is 12 months (9-month intervention period, 3-month follow-up period). The primary data analysis will occur once the last participant has completed the follow-up period, which is anticipated to be on or before July 30, 2022 (if the last participant is recruited on June 28, 2021). The last point of data collection will be completed on or before June 30, 2022 (end of the last participant's follow-up period) and the preceding time will be used to analyze the follow-up data, complete report writing, complete data management (store data securely according to data management plan) and finish the project.

12. FINANCIA/Conflict of interest

No special funding was received for this study, and no conflicts of interest exist in the planning, conduct, or reporting of the study that would affect the results of the study or the interpretation of the results.

13. PUBLICATION POLICY / DISSEMINATION OF RESULTS

The overall results of this research project will be disseminated via journal publication(s), conference presentations, educational seminars. Dr. Eun-Hee Nah will be primarily responsible for taking the lead in publication. All researchers will be acknowledged in publications relating to this project.

14. REFERENCES

1. Younossi ZM, Koenig AB, Abdelatif D, Fazel Y, Henry L, Wymer M. Global epidemiology of nonalcoholic fatty liver disease-meta-analytic assessment of prevalence, incidence, and outcomes. Hepatology. 2016;64(1):73–84. https://doi.org/10.1002/hep.28431 PMID: 26707365

2. Younossi ZM, Golabi P, de Avila L, Paik JM, Srishord M, Fukui N, et al. The global epidemiology of NAFLD and NASH in patients with type 2 diabetes: a systematic review and

meta-analysis. J Hepatol. 2019;71(4):793–801. https://doi.org/10.1016/j.jhep.2019.06.021 PMID: 31279902

3. Atri A, Jiwanmall SA, Nandyal MB, Kattula D, Paravathareddy S, Paul TV, et al. The prevalence and predictors of non-alcoholic fatty liver disease in morbidly obese women - a cross-sectional study from Southern India. Eur Endocrinol. 2020;16(2):152–155. https://doi.org/10.17925/EE.2020.16.2.152 PMID: 33117448

4. Ahmed A, Wong RJ, Harrison SA. Nonalcoholic fatty liver disease review: diagnosis, treatment, and outcomes. Clin Gastroenterol Hepatol. 2015;13(12):2062–2070. https://doi.org/10.1016/j.cgh.2015.07.029 PMID: 26226097

5. Machado MV, Diehl AM. Pathogenesis of nonalcoholic steatohepatitis. Gastroenterology. 2016;150(8):1769–1777. https://doi.org/10.1053/j.gastro.2016.02.066 PMID: 26928243

6. Sanyal AJ, Brunt EM, Kleiner DE, Kowdley KV, Chalasani N, Lavine JE, et al. Endpoints and clinical trial design for nonalcoholic steatohepatitis. Hepatology. 2011;54(1):344–353. https://doi.org/10.1002/hep.24376 PMID: 21520200

7. Acosta A, Streett S, Kroh MD, Cheskin LJ, Saunders KH, Kurian M, et al. White Paper AGA: POWER - Practice Guide on Obesity and Weight Management, Education, and Resources. Clin Gastroenterol Hepatol. 2017;15(5):631–649.e10. https://doi.org/10.1016/j.cgh.2016.10.023 PMID: 28242319

8. Vilar-Gomez E, Martinez-Perez Y, Calzadilla-Bertot L, Torres-Gonzalez A, Gra-Oramas B, Gonzalez-Fabian L, et al. Weight loss through lifestyle modification significantly reduces features of nonalcoholic steatohepatitis. Gastroenterology. 2015;149(2):367–378.e5; quiz e14-e15. https://doi.org/10.1053/j.gastro.2015.04.005 PMID: 25865049

9. Wong VWS, Chan RSM, Wong GLH, Cheung BHK, Chu WCW, Yeung DKW, et al. Community-based lifestyle modification programme for non-alcoholic fatty liver disease: a randomized controlled trial. J Hepatol. 2013;59(3):536–542. https://doi.org/10.1016/j.jhep.2013.04.013 PMID: 23623998

10. Nourian M, Askari G, Golshiri P, Miraghajani M, Shokri S, Arab A. Effect of lifestyle modification education based on health belief model in overweight/obese patients with non-alcoholic fatty liver disease: a parallel randomized controlled clinical trial. Clin Nutr ESPEN. 2020;38:236–241. https://doi.org/10.1016/j.clnesp.2020.04.004 PMID: 32690164

11. Razavi Zade M, Telkabadi MH, Bahmani F, Salehi B, Farshbaf S, Asemi Z. The effects of DASH diet on weight loss and metabolic status in adults with non-alcoholic fatty liver disease: a randomized clinical trial. Liver Int. 2016;36(4):563–571. https://doi.org/10.1111/liv.12990 PMID: 26503843

12. Cai H, Qin YL, Shi ZY, Chen JH, Zeng MJ, Zhou W, et al. Effects of alternate-day fasting on body weight and dyslipidaemia in patients with non-alcoholic fatty liver disease: a randomised controlled trial. BMC Gastroenterol. 2019;19(1):219. https://doi.org/10.1186/s12876-019-1132-8 PMID: 31852444

13. Imajo K, Kessoku T, Honda Y, Tomeno W, Ogawa Y, Mawatari H, et al. Magnetic resonance imaging more accurately classifies steatosis and fibrosis in patients with nonalcoholic fatty liver disease than transient elastography. Gastroenterology. 2016;150(3):626–637.e7. https://doi.org/10.1053/j.gastro.2015.11.048 PMID: 26677985

14. Xiao H, Shi M, Xie Y, Chi X. Comparison of diagnostic accuracy of magnetic resonance elastography and Fibroscan for detecting liver fibrosis in chronic hepatitis B patients: a systematic review and meta-analysis. PLoS One. 2017;12(11):e0186660. https://doi.org/10.1371/journal.pone.0186660 PMID: 29107943

15. Di Martino M, Pacifico L, Bezzi M, Di Miscio R, Sacconi B, Chiesa C, et al. Comparison of magnetic resonance spectroscopy, proton density fat fraction and histological analysis in the quantification of liver steatosis in children and adolescents. World J Gastroenterol. 2016;22(39):8812–8819. https://doi.org/10.3748/wjg.v22.i39.8812 PMID: 27818597

16. Heba ER, Desai A, Zand KA, Hamilton G, Wolfson T, Schlein AN, et al. Accuracy and the effect of possible subject-based confounders of magnitude-based MRI for estimating hepatic proton density fat fraction in adults, using MR spectroscopy as reference. J Magn Reson Imaging. 2016;43(2):398–406. https://doi.org/10.1002/jmri.25006 PMID: 26201284

17. Hamaguchi M, Kojima T, Takeda N, Nakagawa T, Taniguchi H, Fujii K, et al. The metabolic syndrome as a predictor of nonalcoholic fatty liver disease. Ann Intern Med. 2005;143(10):722–728. https://doi.org/10.7326/0003-4819-143-10-200511150-00009 PMID: 16287793

15. APPENDICES

	Baseline	1-M	2-M	3-M	4-M	5-M	6-M
Anthropometric measurement	●	●	●	●			●
Blood test	●			●			●
Ultrasonography and MRE	●						
Health survey	●						●
Lifestyle intervention (1:1 personalized counseling)	●	●	●	●	○	○	●
Self-monitoring					🔵	🔵	

	7-M	8-M	9-M	10-M	11-M	12-M
Anthropometric measurement			●			●
Blood test			●			●
Ultrasonography and MRE						●
Health survey						●
Lifestyle intervention (1:1 personalized counseling)	○	○	●			●
Self-monitoring	🔵	🔵		🔵	🔵	

Figure 1. Overview of Schedule for the study

Abbreviations: M, month; MRE, Magnetic resonance elastograpy.

● : Clinic visit

🔵 ○: Using telephone
