# Supplementary material for: Changes in nonalcoholic fatty liver disease and M2BPGi due to lifestyle intervention in primary healthcare
Source: PLoS One. 2024 Feb 29;19(2):e0298151. doi: 10.1371/journal.pone.0298151 (PMC10903829; doi:10.1371/journal.pone.0298151)
Supplement: S2 File — (PDF) [file pone.0298151.s002.pdf]

연구제목

일차의료기관에서 생활습관중재에 의한 비알콜성지방간의 개선과 M2BPGi 의 변화

1. 일반 사항

1.1 프로토콜 타이틀

일차의료기관에서 생활습관중재에 의한 비알콜성지방간의 개선 및 M2BPGi 의 변화

1.2 이해상충

연구수행을 위한 외부 기관의 지원 없음. 따라서 연구 기획, 수행 및 결과 보고, 결과 해석 및 논문 작성 과정에서 이해상충 발생하지 않음.

1.3 연구수행 책임기관

한국건강관리협회 (Korea Association of Health Promotion, KAHP)

1.4 중재 승인

대한민국 질병관리청 임상연구정보서비스 등록 (KCT0006380)

2. 개요

2.1 제목

일차의료기관에서 생활습관중재에 의한 비알콜성지방간의 개선 및 M2BPGi 의 변화

2.2 목적

비알코올성 지방간의 개선을 위한 개인 맞춤형 중재의 효과 확인

2.3 주요 가설

비알코올성 지방간은 고칼로리 식이와 신체활동 감소와 관련됨. 비알코올성

지방간염을 관리하는 효과적 방법은 질환이 진행되는 것을 예방하는 것이며, 체중 변화에 중점을 둔 생활습관의 변화는 비알코올성 지방간염 관리를 위한 초석임. 체중의 10% 감소를 복합적으로 적용한 생활습관 중재는 간섬유화 뿐 아니라 지방증, 염증을 개선하는데 도움을 줄 수 있음.

2.4. 연구디자인

12 개월 단일표본 중재로, 12 개월 중 9 개월은 중재, 3 개월은 추적 기간임

2.5. 블라인딩/마스킹

식생활 관리 및 신체활동 중재가 핵심적인 사항임, 중재 과정에서 블라인드가 반드시 필요하지 않음. 참여 영양사들이 데이터를 수집하고 분석하는 과정에서 블라인딩 처리 예정.

2.6. 기대효과(OUTCOME)

- 상복부 초음파와 MRE 로 측정한 지방간의 12 개월 후 개선

2.7. 연구기간

12 개월

2.8. 중재

생활습관 중재의 목표는 다음과 같음: 하루 섭취 칼로리 500kcal 감소, 탄수화물을 통해 얻는 에너지를 50~60% 섭취, 지방 섭취 25% 감소, 식이섬유 섭취는 12 g/1,000 kcal

식이상담은 3 일 식사기록에 근거하여 각 대상자별로 식생활분석 프로그램 메디체크 케어플러스(MediCheck Careplus, Computer-Aided Nutritional Analysis program of the Korean Association of Health Promotion)를 활용하여 맞춤형으로 진행되며, 신체활동 증진을 위한 상담은 주당 평균 신체활동 빈도에 기초하여 실시될 예정임.

2.9. 연구 참여자 수

116

2.10. 연구 대상

- 비알코올성 지방간이 있는 20-80 세 성인

2.11. 대상자 모집 및 등록 조건

[대상자 모집 기준]

• 20-80 세 성인

• 대한민국 거주자

• 7 개 건강검진센터에서 건강검진을 받은 사람

• 체질량지수 (BMI) ≥25 kg/m^2

* 상복부 초음파로 최소 경도 지방간으로 진단 받은 경우

[대상자 제외 기준]

* 간질환 진단 (바이러스 간염 등)

* 지방간을 유발할 수 있는 2 차적 원인이 있는 경우

* 과도한 알코올 섭취 이력[>210 g/주 (남성), > 140 g/주 (여성)]

3. 연구 배경

비만과 당뇨병의 증가로 비알코올성 지방간 역시 증가하는 경향을 보이고 있음. 전세계적으로 인구의 약 25% 비알코올성 지방간을 가지고 있는 것으로 보고 되고 있으며 [1], 당뇨 환자의 60% [2] 비만인 사람의 90%가 영향을 미치는 것으로 보고되고 있음 [3]. 비알코올성 지방간의 스펙트럼은 단순 지방증에서 비알코올성 지방간염 (nonalcoholic steatohepatitis, NASH)까지 다양함. 비알코올성 지방간을 가지고 있는 환자들은 특히 비알코올성 지방간염을 동시에 가지고 있는 경우, 간 섬유화로 연결되어 간경화 및 간세포암으로 이어질 수 있음. 비알코올성 지방간을 가지고 있는 사람의 장기적인 결과는 간질환에서의 간섬유화임[4, 5].

비알코올성 지방간의 진행은 고칼로리 식이와 신체활동 감소와 같은 생활습관 요소와 관련되어 있음. 비알코올성 지방간염을 효과적으로 관리하는 것은 이러한 질환의 진행을 막는 것임[6]. 체중조절에 중점을 둔 생활습관 변화는 비알코올성 간염을 관리하는 기본임[7]. 10% 체중 감소를 통한 생활습관 중재는 간섬유화 뿐 아니라, 지방증, 염증을 개선하는데 도움을 줄 수 있음[8]. 최근 대학병원 등에서 이뤄진 일부 연구[8, 9]에서 이런한 중재가 이뤄졌으며, 수주 또는 수개월 동안 이뤄진 엄격하게 조절된 식이에 의한 중재 연구가 보고된 바 있음[10–12].

비알코올성 지방간 개선을 위해 오랜 시간이 소요되는 식이 중재에 연구대상자들이 적응하는 일은 쉽지 않으므로, 지역사회에 기반한 일차보건의료기관에서 생활습관 중재 프로그램이 필요한 시점임.

초음파(ultrasonography, US), MRI(magnetic resonance imaging), and transient elastography 와 같은 영상기술은 간의 지방정도나 경직도를 확인하는데 사용되는 방법임. Magnetic resonance elastography (MRE) 는 간 섬유화 측정에 높은 정확도를 보이는 것으로 보고되고 있음 [13, 14]. MRI 에 기반한 proton-density fat fraction (PDFF) 측정 기술은 간의 지방 수준을 측정하기 위해 개발되었으며, 해당 기술은 모든 간의 영역에서 지방량을 정확하게 측정할 수 있는 것으로 알려져 있음[15, 16]. 그러나 MRI 장비는 고비용이므로, 지역사회 일차보건의료기관을 방문한 개인의 간에서의 지방량과 섬유화 수준을 정기적으로 추척하는데 적용하기는 어려운 제한이 있음.

4. 연구목적/가정

일차보건의료기관에서의 비알코올성 지방간, 대사 요인, M2BPGi (Mac-2-binding protein glycosylated isomer)의 개선을 위한 맞춤형 생활습관 중재의 효과를 확인

5. 중재장소

중재는 유선중재와 대면 중재를 병행하여 진행되며, 유선중재는 핸드폰으로 진행되었고, 클리닉 방문은 건강증진센터 내 비밀이 보장되는 방에서 이뤄질 것임.

6. 연구 계획/연구 디자인

6.1 연구 디자인

12 개월 단일 중재 연구.

6.2 모집단, 표본 사이즈

선행연구에서 1-2 년 이내 지방간이 개선되는 비율이 16%로 보고됨 [17]. 현 중재에서 연구대상자의 지방간이 개선되는 비율은 선행 연구의 2 배인 32%고 가정하였으며, 90% power (α=0.05, 1–β=0.9), 5% significance level, 추적조사 시 탈락되는 비율을 30%로 가정하고, chi-square test 로 산출하였을 때 총 116 명의 연구대상자 수가 요구되었음.

6.3 통계분석

SAS software (version 9.4, SAS Institute, Cary, NC, USA)를 활용하여 통계 분석하며, 사전 시점과 12 개월 시점 간의 임상변수 간의 repeated-measures ANOVAs 와 chi-square tests 로 분석할 것임. Post-hoc comparisons 는 Bonferroni adjustment for multiple comparisons 로 진행할 것임. 사전시점과 12 개월의 차이는 Cochran's Q test 로 분석하며, 12 개월 시점에서의 비알코올성 지방간의 개선 그룹과 미개선 그룹 간의 차이는 paired *t*-tests 로 확인할 것임. Multivariate logistic regression analyses 이 12 개월 후 비알코올성 지방간 개선에 영향을 미치는 요인을 확인하기 위해 적용되며, PDFF and

M2BPGi levels 간의 관견성은 Pearson 의 상관관계 분석으로 확인할 것음. 모든 통계적 유의성은 $P<0.05$ 를 기준으로 함.

6.4 대상자 모집 및 연구대상자 선정 (고지된 동의 포함)

연구참여자는 대한민국의 5 개 시의 7 개 건강검진센터에서 모집되며, 연구팀의 전화로 초기 대상자의 중재 참여 의사를 확인할 것임. 해당 대상자가 본 중재에 참여 가능한 대상자 인지 확인하기 위해 다방면의 조사를 실시하고, 이를 위해 조사자들은 대상자의 포함/제외 조건을 숙지할 예정임. 잠재적으로 연구 참여가 가능한 대상자들에게 추가적인 검사 정보나 주요 반응 등에 대해 설명하고, 첫번째 방문을 위한 스케쥴을 확인할 것임. 해당 시점에서 참여자들은 세부적인 중재 프로그램에 대한 안내를 받으며, 본 연구팀은 충분한 설명 후 연구대상자의 중재 참여를 위한 고지된 동의를 취득할 예정임.

6.5 생활습관 중재 기간

생활습관 중재는 2021 년 7 월 2 일부터 2022 년 7 월 30 일까지 진행될 것이며, 7 개 센터의 임상영양사들은 표준화된 중재 운영을 위해 매뉴얼을 제공 받고, 운영 프로토콜에 대한 사전 교육을 받을 예정임. 연구대상자들은 6 회 건강증진센터를 방문(baseline, 1, 2, 3, 6, 9, 12 개월) 하고, 센터 방문 이외 유선 중재와 셀프모니터링(4, 5, 7, 8 개월)을 9 개월 시점까지 받을 예정임.

6.5.1 식이 중재

개별화된 맞춤형 상담이 연구대상자의 3 일 식사 기록에 기반하여 실시될 것임. 생활습관 중재는 일일 에너지 섭취 기준에 따라 탄수화물을 통해 얻는 에너지를 50~60%만 섭취하고, 지방 섭취 25% 감소, 식이섬유 섭취는 12 g/1,000 kcal 를 목표로 함. 식이상담은 3 일 식사기록에 근거하여 각 대상자별로 식생활분석 프로그램 메디체크 케어플러스(MediCheck Careplus, Computer-Aided Nutritional Analysis program of the Korean Association of Health Promotion)를 활용하여 맞춤형으로 진행될 것임.

6.5.2 운동 중재

신체활동을 증진시키기 위한 맞춤형 상담은 주당 평균 운동량에 근거하여 실시될 것임. 매 방문 시 참여자들은 단축형 국제신체활동조사(the short-form International Physical Activity Questionnaire)에서 지난 7 일간의 활동량을 기준으로 한 그들의 신체활동량을 증가시키기 위한 개별화된 조언을 받을 것임. IPAQ 에서는 주당 5 일 이상 최소 30 분 이상 중강도 신체활동 혹은 주당 150 분의 중강도 신체활동을 권장하며, 중강도 신체활동에는 걷기, 수영, 자전거타기 등이 권장되고 있음. 신체활동의 종류, 지속기간, 빈도 등은 참여자의 생활습관이나 건강상태에 따라 개별 맞춤형으로 권장될 것임.

6.6 추적조사

집중 중재가 이뤄지는 기간이 기록될 것이나, 참여자들은 3 개월의 추적조사 기간동안 셀프 모니터링을 하게 되며, 그 결과를 영양사들이 확인할 것임. (Figure 1).

6.7 중재 및 추적조사 일정

중재 및 추적조사 일정은 Figure 1 에 기록되어 있음. .

6.8 동의획득 절차

잠재적인 연구참여자를 대상으로 연구 참여에 관심을 표명한 사람을 대상으로 연구자들이 1 차적인 연락을 할 예정이며(이메일, 핸드폰 등), 모든 참여자들은 모든 조사를 완료해야 함. 모든 잠재적 참여자들을 대상으로 고지된 동의를 받을 예정임.

7. 윤리적 고려

소속기관에서 연구수행의 윤리적 수행에 대해 승인을 획득할 예정임.

7.1 자발적 대상자 모집

연구대상자 모집 방법은 6.4 에 제시되어 있음. 연구대상자 모집은 연구자들에 의해, 수검자를 대상으로 동의를 받고 전향적으로 진행되므로 대상자 모집 과정에서 실제적, 잠재적 위험은 존재하지 않음.

7.2 연구결과의 기밀성 보장

전반적인 연구결과가 학술지 출판, 컨퍼런스 발표, 교육 세미나 등에서 발표될 예정이며, 결과는 평균 등으로 보고되어, 개인을 식별할 수 없는 형태로 보고될

예정임. 개인의 데이터가 필요할 경우, 특정 사람을 지칭하는 것이 "한 사람" 등의 형태로 보고될 예정이며, 개인을 확인하기 위해 개인정보가 삭제된 ID 를 활용할 예정임.

7.3 연구 참여에 따른 잠재적 위험

7.3.1 불편감, 감염 위험

채혈로 인해 약간의 불편감이나 감염, 채혈 부위의 멍이 들 가능성이 있으나, 프로토콜에 따라 진행되어 신체적 위험은 최소화될 것임. 본 연구는 전형적인 건강검진 참여자를 대상으로 하므로, 건강검진에 따른 일반적인 신체적 위험 정도의 위험만 있을 것으로 판단됨

7.3.2 사회적 스트레스

연구 참여에 따라 식이 세션, 연구일정에 따른 건강증진센터 방문 등이 있을 수 있으며, 이에 따른 시간이 소요되는 부분이 발생됨. 참여자들의 다빈도 방문에 따른 부담을 경감시키기 위해, 적절한 시간이 세션이 소요되도록 할 예정임.

8. 안전에 대한 고려

8.1 연구참여에 따른 부작용

연구의 참여로 인해 연구대상자들이 직접적으로 받는 모든 부작용은 기록될 예정임. 본 중재는 식생활을 중심으로 한 생활습관 중재이므로, 예상되는 심각한 부작용은

매우 낮은 상황이나, 연구대상자들은 중재 과정에서 발생되는 어떤 부작용도 연구 영양사와 의사들에 의해 직접 조언, 처치를 받을 수 있음

9. 기대되는 결과

모든 주요 기대결과 및 부차적인 기대결과는 Figure 1 에 기록되어 있음. Figure 1 은 각 연구대상자를 위해 중재 참여 기간 동안 시점별로 측정되는 항목 등이 정리되어 있음..

9.1 Primary outcome

- 중재 12개월 후 상복부 초음파와 간 MRE에 의해 최소한 1단계 이상 비알코올성 지방간 정도의 개선

9.2 Secondary outcome

- 중재 12개월 후 대사요소와 M2BPGi 수준의 개선

  - 체질량지수 (BMI) (kg/m^2)
  - 허리둘레 (cm
  - 혈압(mmHg)
  - 생화학 검사: 간기능, 지질, 식전혈당, 인슐린 수준

10. 데이터 관리

10.1 데이터 수집

다음 데이터 항목을 수집할 예정임

- 설문조사 (승인된 양식)
- 한국건강관리협회 건강검진에서 이뤄지는 병리학 및 영상진단 결과
- 연구 영양사와 매 중재 방문 시점에서 획득되는 측정 자료: 체중, 허리둘레, 혈압, 설문조사
- 연구 참여자에 의해 이뤄지는 셀프 모니터링 자료: 체중, 식이 데이터
- 연구 시점별로 연구대상자에게 제공되는 관련 자료(부작용 등)

10.2 데이터 저장

10.1 에 언급된 바와 같이 모든 연구참여자의 수집된 데이터는 한국건강관리협회 DB 에 저장 및 보관됨

10.3 데이터 사용

관련된 데이터는 한국건강관리협회 DB 에서 개인정보를 제외한 엑셀 파일 형태로 추출될 예정이며, 모든 데이터는 연구 분석을 위한 결합된 형태로 연구 기간동안 수집되고 활용될 예정임. 데이터는 연구 기간동안 연구자들에 의해 비밀번호로 보호된 컴퓨터에 보관되며, 분석될 예정임.

11. 타임라인

연구자들은 2021 년 6 월 3 일에 본 연구를 시작하고 13 개월 후인 2022 년 7 월

30 일에 종료할 예정임. 2021 년 6 월 3 일에 대상자 모집을 시작하여, 1 달 후인 2021 년 6 월 28 일까지 116 명의 연구대상자 모집을 종료할 것임. 연구참여자들이 중재에 참여하는 시점은 모집된 시점이 달라 모두 상이하며, 추적조사 기간을 포함하여 각 연구대상자들이 중재에 총 참여하는 시간은 총 12 개월임(9 개월 중재, 3 개월 추적조사 기간). 주요 분석를 위한 데이터는 마지막 참여자의 추적조사가 종료되는 시점에 추출되며, 해당 시점은 2022 년 7 월 30 일로 예상되고 있음. 데이터 수집은 2022 년 6 월 30 일 이전에 종료되며, 추적조사 데이터의 분석, 보고서 작성, 데이터 관리와 프로젝트 종료 등에 시간이 추가로 소요될 예정임.

12. 재정/이해상충

연구수행을 위한 외부 기관의 지원 없었음. 따라서 연구 기획, 수행 및 결과 보고, 결과 해석 및 논문 작성 과정에서 이해상충 발생하지 않음.

13. 출판 및 연구결과 보도에 따른 정책

본 연구의 전반적인 결과는 학술지 출판, 회의 발표, 교육 세미나 등에서 발표될 수 있음. 나은희 연구책임자가 출판을 주도할 것이며, 모든 연구자들이 출판과 관련하여 역할을 다할 것임.

14. 참고문헌

1. Younossi ZM, Koenig AB, Abdelatif D, Fazel Y, Henry L, Wymer M. Global epidemiology of nonalcoholic fatty liver disease-meta-analytic assessment of prevalence, incidence, and outcomes. Hepatology. 2016;64(1):73–84. https://doi.org/10.1002/hep.28431 PMID: 26707365

2. Younossi ZM, Golabi P, de Avila L, Paik JM, Srishord M, Fukui N, et al. The global epidemiology of NAFLD and NASH in patients with type 2 diabetes: a systematic review and meta-analysis. J Hepatol. 2019;71(4):793–801. https://doi.org/10.1016/j.jhep.2019.06.021 PMID: 31279902

3. Atri A, Jiwanmall SA, Nandyal MB, Kattula D, Paravathareddy S, Paul TV, et al. The prevalence and predictors of non-alcoholic fatty liver disease in morbidly obese women - a cross-sectional study from Southern India. Eur Endocrinol. 2020;16(2):152–155. https://doi.org/10.17925/EE.2020.16.2.152 PMID: 33117448

4. Ahmed A, Wong RJ, Harrison SA. Nonalcoholic fatty liver disease review: diagnosis, treatment, and outcomes. Clin Gastroenterol Hepatol. 2015;13(12):2062–2070. https://doi.org/10.1016/j.cgh.2015.07.029 PMID: 26226097

5. Machado MV, Diehl AM. Pathogenesis of nonalcoholic steatohepatitis. Gastroenterology. 2016;150(8):1769–1777. https://doi.org/10.1053/j.gastro.2016.02.066 PMID: 26928243

6. Sanyal AJ, Brunt EM, Kleiner DE, Kowdley KV, Chalasani N, Lavine JE, et al. Endpoints and clinical trial design for nonalcoholic steatohepatitis. Hepatology. 2011;54(1):344–353. https://doi.org/10.1002/hep.24376 PMID: 21520200

7. Acosta A, Streett S, Kroh MD, Cheskin LJ, Saunders KH, Kurian M, et al. White Paper AGA: POWER - Practice Guide on Obesity and Weight Management, Education, and Resources. Clin Gastroenterol Hepatol. 2017;15(5):631–649.e10. https://doi.org/10.1016/j.cgh.2016.10.023 PMID: 28242319

8. Vilar-Gomez E, Martinez-Perez Y, Calzadilla-Bertot L, Torres-Gonzalez A, Gra-Oramas B,

Gonzalez-Fabian L, et al. Weight loss through lifestyle modification significantly reduces features of nonalcoholic steatohepatitis. Gastroenterology. 2015;149(2):367–378.e5; quiz e14-e15. https://doi.org/10.1053/j.gastro.2015.04.005 PMID: 25865049

9. Wong VWS, Chan RSM, Wong GLH, Cheung BHK, Chu WCW, Yeung DKW, et al. Community-based lifestyle modification programme for non-alcoholic fatty liver disease: a randomized controlled trial. J Hepatol. 2013;59(3):536–542. https://doi.org/10.1016/j.jhep.2013.04.013 PMID: 23623998

10. Nourian M, Askari G, Golshiri P, Miraghajani M, Shokri S, Arab A. Effect of lifestyle modification education based on health belief model in overweight/obese patients with non-alcoholic fatty liver disease: a parallel randomized controlled clinical trial. Clin Nutr ESPEN. 2020;38:236–241. https://doi.org/10.1016/j.clnesp.2020.04.004 PMID: 32690164

11. Razavi Zade M, Telkabadi MH, Bahmani F, Salehi B, Farshbaf S, Asemi Z. The effects of DASH diet on weight loss and metabolic status in adults with non-alcoholic fatty liver disease: a randomized clinical trial. Liver Int. 2016;36(4):563–571. https://doi.org/10.1111/liv.12990 PMID: 26503843

12. Cai H, Qin YL, Shi ZY, Chen JH, Zeng MJ, Zhou W, et al. Effects of alternate-day fasting on body weight and dyslipidaemia in patients with non-alcoholic fatty liver disease: a randomised controlled trial. BMC Gastroenterol. 2019;19(1):219. https://doi.org/10.1186/s12876-019-1132-8 PMID: 31852444

13. Imajo K, Kessoku T, Honda Y, Tomeno W, Ogawa Y, Mawatari H, et al. Magnetic resonance imaging more accurately classifies steatosis and fibrosis in patients with nonalcoholic fatty liver disease than transient elastography. Gastroenterology. 2016;150(3):626–637.e7. https://doi.org/10.1053/j.gastro.2015.11.048 PMID: 26677985

14. Xiao H, Shi M, Xie Y, Chi X. Comparison of diagnostic accuracy of magnetic resonance

elastography and Fibroscan for detecting liver fibrosis in chronic hepatitis B patients: a systematic review and meta-analysis. PLoS One. 2017;12(11):e0186660. https://doi.org/10.1371/journal.pone.0186660 PMID: 29107943

15. Di Martino M, Pacifico L, Bezzi M, Di Miscio R, Sacconi B, Chiesa C, et al. Comparison of magnetic resonance spectroscopy, proton density fat fraction and histological analysis in the quantification of liver steatosis in children and adolescents. World J Gastroenterol. 2016;22(39):8812–8819. https://doi.org/10.3748/wjg.v22.i39.8812 PMID: 27818597

16. Heba ER, Desai A, Zand KA, Hamilton G, Wolfson T, Schlein AN, et al. Accuracy and the effect of possible subject-based confounders of magnitude-based MRI for estimating hepatic proton density fat fraction in adults, using MR spectroscopy as reference. J Magn Reson Imaging. 2016;43(2):398–406. https://doi.org/10.1002/jmri.25006 PMID: 26201284

17. Hamaguchi M, Kojima T, Takeda N, Nakagawa T, Taniguchi H, Fujii K, et al. The metabolic syndrome as a predictor of nonalcoholic fatty liver disease. Ann Intern Med. 2005;143(10):722–728. https://doi.org/10.7326/0003-4819-143-10-200511150-00009 PMID: 16287793

15. 부록

	Baseline	1-M	2-M	3-M	4-M	5-M	6-M
신체계측	●	●	●	●			●
혈액검사	●			●			●
영상검사 (상복부 초음파 및 간 MRE)	●						
설문조사	●						●

1:1 맞춤형 생활습관 중재	●	●	●	●	○	○	●
셀프모니터링					🔵	🔵	

	7-M	8-M	9-M	10-M	11-M	12-M
신체계측			●			●
혈액검사			●			●
영상검사 (상복부 초음파 및 간 MRE)						●
설문조사						●
1:1 맞춤형 생활습관 중재	○	○	●			●
셀프모니터링	🔵	🔵		🔵	🔵	

Figure 1. 연구 스케쥴

Abbreviations: M, month; MRE, Magnetic resonance elastograpy.

● : 클리닉 방문 중재

🔵 ○: 유선 중재
