# Supplementary material for: Changes in nonalcoholic fatty liver disease and M2BPGi due to lifestyle intervention in primary healthcare
Source: PLoS One. 2024 Feb 29;19(2):e0298151. doi: 10.1371/journal.pone.0298151 (PMC10903829; doi:10.1371/journal.pone.0298151)
Supplement: S2 Table — (PDF) [file pone.0298151.s004.pdf]

Table A. The number of participants in each grade of fatty liver using ultrasonography at baseline and 12-month.

		After 12 month				Total
		Normal	Mild	Moderate	Severe	
Baseline	Normal	0	0	0	0	0
	Mild	**8**	20	7	0	35
	Moderate	**1**	**21**	43	1	66
	Severe	**1**	**2**	**1**	3	7
Total		10	43	51	4	108

Thick letters indicate participant numbers of fatty liver improving after 12 months.

Table B. The lifestyle behavior characteristics of participants at baseline and follow-up times.

	Baseline	After 6 month (T6)	After 12 month (T12)	*P*	Post hoc†
Physical Activity, IPAQ					
Low	26 (24.1)	15 (14.3)	14 (13.6)	0.024	
Moderate	56 (51.9)	57 (54.3)	43 (41.8)		
High	26 (24.1)	33 (31.4)	46 (44.7)		
Mean±SD	2186.0 ± 2201.3	2670.3 ± 2572.0	3302.1 ± 2843.4	<0.001	T0<T6, T6<T12
Diet, MDA					
Good	13 (12)	8 (7.5)	6 (5.6)	<0.001	
Fair	60 (55.6)	38 (35.5)	44 (40.7)		
Poor	35 (32.4)	61 (57)	58 (53.7)		
Mean±SD	30.7 ± 6.8	27.1 ± 6.7	27.5 ± 6.6	<0.001	T0>T6

Data are mean±standard deviation or *n* (%) values.

Abbreviation: IPAQ, international physical activity questionnaire; SD, standard deviation; MDA, mini dietary assessment.

†Post-hoc comparisons were performed using Bonferroni adjustment for multiple comparisons.
